# *Mycobacterium tuberculosis* F-ATP Synthase Inhibitors and Targets

**DOI:** 10.3390/antibiotics13121169

**Published:** 2024-12-03

**Authors:** Amaravadhi Harikishore, Gerhard Grüber

**Affiliations:** School of Biological Sciences, Nanyang Technological University, 60 Nanyang Drive, Singapore 637551, Singapore

**Keywords:** F-ATP synthase, OXPHOS, ETC (electron transport chain), inhibitors, mycobacteria, bedaquiline

## Abstract

*Mycobacteria tuberculosis* (*Mtb*) infection causes tuberculosis (TB). TB is one of the most intractable infectious diseases, causing over 1.13 million deaths annually. Under harsh growing conditions, the innate response of mycobacteria is to shut down its respiratory metabolism to a basal level, transit into a dormant, non-replicating phase to preserve viability, and establish latent infection. *Mtb* utilizes non-canonical regulatory mechanisms, such as alternative oxidase pathways, to survive in low oxygen/nutrient conditions. The bacterium’s survival in its native microenvironmental niches is aided by its ability to evolve mutations to drug binding sites, enhance overexpression of various enzymes that activate β-lactam antibiotics hydrolysis, or stimulate efflux pathways to ward off the effect of antibiotics. Bedaquiline and its 3,5-dialkoxypyridine analogs, sudapyridine and squaramide S31f, have been shown to be potent *Mtb* F_1_F_O_-ATP synthase inhibitors of replicating and non-replicating *Mtb* and have brought oxidative phosphorylation into focus as an anti-TB target. In this review, we attempt to highlight non-canonical structural and regulatory pathogen-specific epitopes of the F_1_-domain, ligand development on such sites, structural classes of inhibitors targeting the Fo-domain, and alternative respiratory metabolic responses that *Mtb* employs in response to bedaquiline to ensure its survival and establish latent infection.

## 1. Introduction

**Figure 1:**
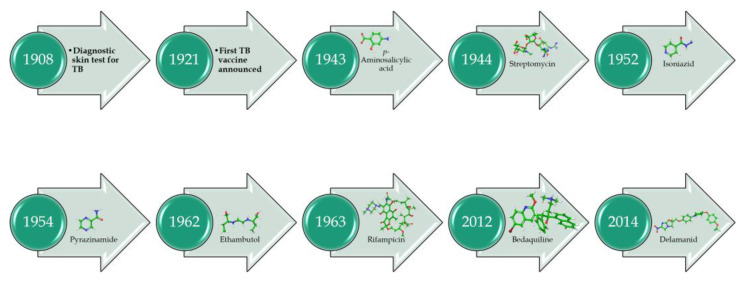
A historical journey of TB vaccine and drug discovery.

Following the successful isolation of the causative agent of tuberculosis (TB) *Mycobacterium tuberculosis* (*Mtb*) in 1882 [1], the development of TB diagnosis in 1908, the first vaccination in 1920–21 [2], the first anti-TB drugs *p*-aminosalicylic acid [3] and streptomycin [4] in the 1940s (Figure 1), followed by the discovery of the first-line anti-TB drugs isoniazid (INH) [5], pyrazinamide (PZA) [6], ethambutol [7], and rifampicin [8], and the more recently developed bedaquiline (BDQ) [9] and delamanid [10], TB continues to be a leading infectious disease with a global estimate of 1.13 million deaths annually among HIV-negative people [1]. Unfortunately, the global incidence of TB has recently resurged to levels not seen since 2019, with an estimate of about 10.6 million new infections [11]. Global efforts to end TB by 2025 have only shown a marginal 19% net reduction from 2015–2022 and are far off from the 2025 milestone for reducing TB infections to less than 0.6 million [11]. *Mtb* infections are spread by tuberculoid bacilli-infested aerosol during coughing or liquid droplets from the runny nose of infected patients [12]. The incidence of multidrug-resistant TB (MDR-TB) that lacks sensitivity to first-line therapeutic drugs such as INH, Rifampicin (R), or PZA [11]. Further, the emergence of extremely drug-resistant strains that contribute to latent TB infections makes it exceedingly difficult to control and treat TB [11].

**Figure 2:**
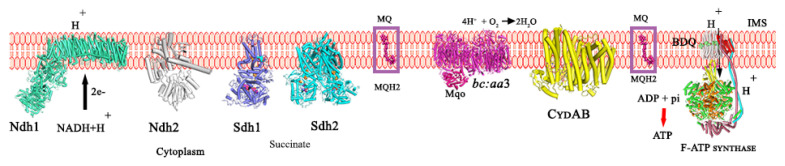
Mycobacterial oxidative phosphorylation enzymes. The mycobacterial proton-translocating Ndh-1 enzyme utilizes NADH as substrate during aerobic growth or non-proton contributing Ndh-2 during hypoxia. Likewise, Sdh1 and Sdh2 use their substrate succinate and transfer electrons to maintain the menaquinone (MQ/MQH_2_) pool. Menaquinol and Mqo transfer the electrons to the cytochrome *bcc/aa_3_* oxidase and/or the cytochrome *bd* oxidase. The proton gradient thus generated, triggers proton translocation from the intermembrane space to the cytoplasm via the two half channels of the subunit *a*-*c_9_*-ring interface and induces conformational changes in the F_1_-sector to stimulate ATP synthesis.

*Mtb* is able to re-route its metabolic respiration depending on the presence or absence of oxygen and nutrients and maintains a very basal level of respiration. This enables the pathogen to switch into a dormant phase, thereby surviving the adverse growth conditions prevailing in phagosomes or granulomas and sustaining its life until such time as favorable growing conditions are encountered [13,14]. Such dynamic adaptation to adverse growing conditions achieved by lowering the consumption of the main energy fuel, adenosine triphosphate (ATP), and maintaining ATP/ADP homeostasis is conferred by the oxidative phosphorylation (OXPHOS) pathway, including the enzymes of the electron transport chain (ETC) and the final catalyst, the F_1_F_O_-ATP synthase (Figure 2) [15]. The NADH dehydrogenase (NDH-1 and/or NDH-2), the succinate dehydrogenase (Sdh-1 or Sdh-2) and the malate/quione oxidoreductase (Mqo) help in recycling the central metabolic electron carrier NADH and the oxidation of the Krebs cycle substrates succinate and malate, respectively, to transfer electrons to the menaquinone (MQ) pool [16,17,18]. The cytochrome *bcc/aa_3_* oxidase supercomplex and the cytochrome *bd* oxidase accept electrons from menaquinol and efficiently reduce oxygen to water [19,20,21]. While electrons are transferred along the ETC, protons are translocated from the cytoplasm to the intermembrane space to generate the proton motive force (pmf). These protons are transported back to the cytoplasm by the membrane-embedded F_O_-domain of the F_1_F_O_-ATP synthase, providing the necessary torque to induce ATP synthesis in the F_1_-domain of this engine [22].

**Figure 3:**
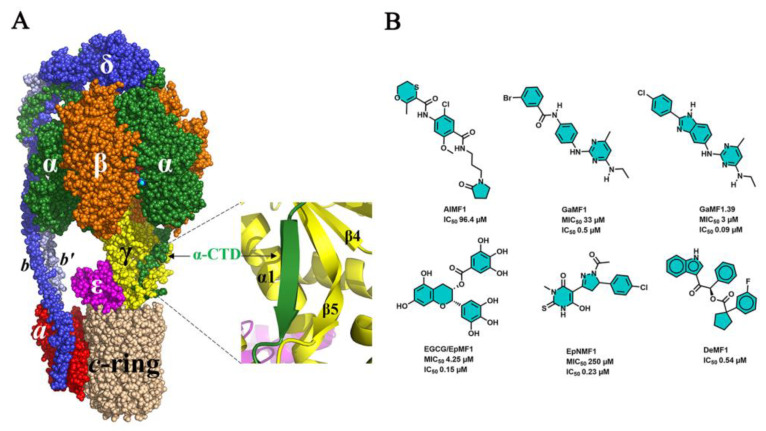
Structure and inhibitors of the mycobacterial F-ATP synthase. (**A**). Cryo-EM structure of the *Mtb* F-ATP synthase (PDB ID: 8j0S [26]) and a zoom-in, showing the β-sheet interaction of α’s C-terminal extension (green) with a β5-sheet of the rotary subunit γ (yellow). (**B**). Inhibitors that target the mycobacterial F-ATP synthase subunit α (AlMF1), γ (GaMF1, GaMF1.39), ε (EGGC/EpMF1, EpNMF1), and δ (DeMF1).

The mycobacterial F_1_F_O_-ATP synthase consists of the membrane-bound proton-translocating F_O_-part and a catalytic F_1_-sector (Figure 3A) [23,24,25,26]. The F_O_-domain is formed by the membrane-embedded subunits *a:c_9_*, forming two half-proton channels, which translocate the protons from the intermembrane space to the cytoplasm. The rotary motion of the *c_9_*-ring during proton transport drives the rotation of the central stalk subunits γ:ε, which couple proton transport with ATP synthesis from ADP + P_i_ in the catalytic α_3_β_3_-headpiece of the soluble F_1_-segment. The α_3_β_3_ and *a:c_9_* domains are connected by the peripheral stalk subunits *b*-δ:*b′*, which enable smooth transmission of power between the rotary *c_9_*-ring turbine and the α_3_:β_3_:γ:ε domain [25]. 

As an obligate aerobe, *Mtb* is dependent on OXPHOS [27]. Although equipped with an ETC that is highly branched with NDH, Sdh isoforms, and alternative oxidases, only one F-ATP synthase form exists in the pathogen (Figure 2). *Mtb* can survive with the deletion of one of the ETC isoforms or oxidases under oxygen conditions [17,28]. In contrast, the *Mtb* F-ATP synthase is essential for the bacterium [16]. CRISPR interference studies employing transcriptional knockdown of *Mtb’s a*-subunit (atpB gene)/*c*-subunit (atpE) genes have demonstrated that they are paramount for the survival of the pathogen [29]. Knockdown of both (atpB/atpE genes) strains with a single guide (sg) RNA prevented growth with a potent minimum inhibitory concentration 99 (MIC_99_) in the range of 4–12 ng/mL [29]. Among these two genes, atpE depletion was shown to be bactericidal (1.0 log_10_ CFU/mL at 10 ng/mL) by day 5, while the atpB knockdown caused <1 log_10_ CFU/mL reduction at 10–100 ng/mL and needed a higher concentration of 300 ng/mL for cidal effects (1.7 log_10_ CFU/mL) by day 5. The discovery of bedaquiline (BDQ) with killing potency against replicating and non-replicating *Mtb* underscored the importance of the *Mtb* F-ATP synthase as an important anti-TB target [9].

In this review, we discuss the non-canonical modes of mycobacterial F-ATP synthase regulation by targeting the unique pathogen-specific epitopes, describe *Mtb’s* metabolic responses to inhibitors, and highlight the structural classes of ligands targeting the *Mtb* F-ATP synthase engine. These have been discussed in context with the most recent atomic structures of the inhibitor-bound *Mtb*- and human mitochondrial F-ATP synthase, paving the way to design improved drugs with low toxicity.

## 2. Unique Epitopes and Inhibitors Binding to the Mycobacterial F_1_-Domain

### 2.1. The Regulatory Element of Mtb Subunit α

In comparison with canonical α-subunits, the *Mtb* counterpart harbors a unique C-terminus [30,31] with a prokaryotic ubiquitin-like protein (PUP) site at residue K489 [32], as well as multiple lysine residues at its C-terminus. This PUP K489 and the trail of C-terminal lysine residues could anchor proteasomal degradation [32]. Strikingly, *Mtb’s* subunit α also possesses a unique C-terminal domain (CTD; amino acids 521–540) [31], which mediates the suppression of ATP hydrolysis activity, thereby contributing to the phenomenon of latent ATP hydrolysis, prevention of ATP wastage, and ATP/ADP homeostasis [30,31]. This extension is unstructured and becomes partially folded in one of the three α’s, whose CTD interacts with a *Mtb*-specific sequence of the rotary subunit γ [23,24,25,26], resulting in intramolecular friction [14]. The recent cryo-EM structure of the *Mtb* F-ATP synthase visualizes how the CTD becomes locked by forming a parallel β-sheet with a β5-strand of subunit γ (Figure 3A) [26]. Deletion of the CTD enhanced ATP hydrolysis by 16.5-fold in comparison to the wild-type (WT) enzyme. As anticipated, this deletion showed a modest decrease in ATP synthesis (12%) in comparison to WT enzyme [25].

Additional genetic, structural, and biochemical insights opened the door for a protein-based pharmacophore model, virtual screening, and characterization of the novel inhibitor N-(2-chloro-5-methoxy-4-((3-(2-oxopyrrolidin-1-yl)-propyl)carbamoyl)phenyl)-2-methyl-5,6-dihydro-1,4-oxathiine-3-carboxamide (Figure 3B), called AlMF1 [33]. The inhibitor reduces NADH-driven ATP synthesis of mycobacterial-inverted membrane vesicles (IMVs) and the recombinant and reconstituted F_1_F_O_-ATP synthase in the µM range [33].

Interestingly, gene knockout studies have revealed that protein levels of subunit α could be negatively regulated by upstream inhibition of the *Mtb* phosphatase enzyme (ptpA). The mutant strain was characterized as having low ATP synthesis and growth inhibition in nonpathogenic *Mtb* H37Ra [34]. These results might warrant further investigation into the mapping of the biophysical interaction of these two enzymes and how these or additional unknown phosphatase motifs may be involved in mediating the inhibition of *atpA* protein levels.

### 2.2. Role of the Extra Loop in Subunit γ

The mycobacterial γ-subunit harbors a unique 14 amino acid domain, whose deletion decreases ATP synthesis while increasing ATP hydrolysis [35]. The latter enables ATP-driven proton-pumping, thereby affecting the membrane potential [36]. These data highlighted the importance of latent ATPase activity of the mycobacterial F-ATP synthase in keeping the pmf at a level sufficient for ATP formation as well as activating pmf-driven in- and efflux pumps [36]. Employing this segment as a selective mycobacterial epitope for structure-based drug design, GaMF1 (Figure 3B) has been identified as a potent anti-TB inhibitor targeting the *Mtb* γ-loop and the *c*-ring interface [37]. The inhibitor also shows immediate killing potency against resistant clinical isolates (isoniazid or rifampicin and isoniazid plus rifampicin-resistant *Mtb* strains) [37]. Further medicinal chemistry efforts on GaMF1 resulted in the lead molecule GaMF1.39 (Figure 3B) with a MIC_50_ of 3 µM against *Mtb* [37]. GaMF1.39, in combination with OXPHOS enzyme inhibitors such as clofazimine (CFZ), Q203 (Telacebec), and ND-011992, displayed improved potency in intracellular ATP levels and bactericidal effects in *Mtb*-infected macrophages [38].

### 2.3. The N- and C-Terminal Domain of ε Are Important for Coupling

**Figure 4:**
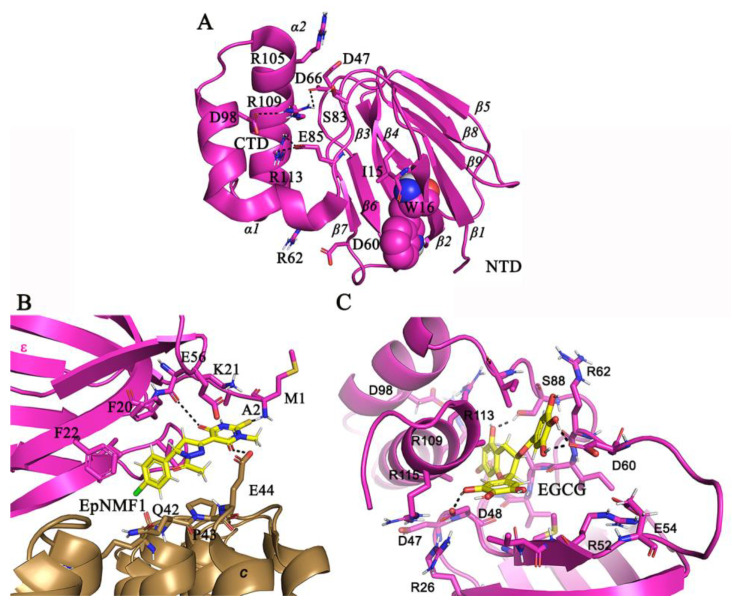
*Mtb* ε structure, highlighting the important interaction sites. (**A**) Highlighted are the polar interactions between the N-terminal domain (NTD) and CTD. The W16 residue (shown in spheres, pdb: 5yio [30]), whose mutation induces hypersusceptibility to BDQ, is highlighted. (**B**) EpNMF1 interaction with ε’s N-terminal residues K21, F20, F22, A2. (**C**) Binding interactions of EGCG at the C-terminal α_2_-helix and polar contacts with D48, D60, S88 with hydroxy groups of EGCG.

Together with subunits α and γ, the *Mtb* subunit ε subunit (Figure 4A) is one of the endogenous ATP hydrolysis inhibitors. Its β-barrel domain transfers the rotary motion of the *c_9_*-ring to the catalytic α_3_β_3_-headpiece [39], whereby the N-terminal residues M1–L4, which are near the loop region of the *c_9_*-ring, play a critical role in the stability of the enzyme [40]. Based on these insights, in silico studies discovered the novel inhibitor EpNMF1 (Figure 3B), which docks to the N-terminal ε residues M1–A2, F22, F24, anchors to the *c*-ring loop-residues (Figure 4B), and inhibits ATP synthesis with a potency of about 0.2 µM [40]. These findings underscored that ε’s N-terminal domain is an attractive inhibitor target.

Furthermore, the N-terminal residues such as A10-W16, L61-64, and A81-I90 and the C-terminal amino acids A112-R113 (Figure 4C) were revealed to be involved in inter-domain contacts [39]. Mutagenesis of W16A led to a considerable reduction in ATP synthesis, enhancement in ATP hydrolysis of IMVs, and enhanced sensitivity to BDQ. Overexpression of WT ε has abrogated the sensitivity to BDQ, further supporting the notion that dysregulation of ε functioning could synergize the MIC potency of BDQ [41]. Importantly, a quadruple ε R105A, R111A, R113A, and R115A mutant strain revealed diminished whole-cell ATP synthesis, defective colony morphology, and dysregulated oxidative phosphorylation [42]. Virtual screening using the quadruple mutant data led to the characterization of epigallocatechin gallate (EGCG, Figure 3B and Figure 4C) as a potent binder to subunit ε [42]. EGCG inhibited both the NADH and succinate-driven ATP synthesis with half-maximum inhibitory concentrations (IC_50_) of 155 nM and 2.2 µM, respectively [32]. Raju, A. et al. [43] showed that EGCG, catechin, epicatechin, and gallic acid potently inhibit the growth of *Mtb* H_37_Rv cultures, with EGCG being the most potent one (MIC_50_ = 4.25 µM) [43]. EGCG-trehalose coated microsphere formulation (25 mg) displayed dose and time-dependent killing of *Mtb* inside mouse macrophages by targeting additional host pathways such as the induction of lysosome acidification and autophagy. Further, in vivo studies using the microencapsulated EGCG inhalation dosed at 50 mg, administered 5 days/week for 6 weeks, showed reduced bacterial burden up to 2.54 Log_10_ CFU after six weeks of treatment in mice [44].

### 2.4. Targeting δ’s Elastic Energy Transmission 

The mycobacterial subunit δ contains a unique 111 amino acid N-terminal extension, which, together with δ’s central part, is involved in movements of subunit δ, which enable coupling of elastic energy generated by *c*-ring revolution and γ:ε rotation [25,45,46]. Mutational studies on the recombinant enzyme, including residues (δR171, δR171G, δR177Q, and δQ178R) proposed to be involved in the movements and located close to the catalytic α_3_:β_3_ headpiece, underscored the importance of δ for ATP synthesis [46]. Based on these observations, in silico approaches identified DeMF1 (2–(1H-indol–3–yl)–2–oxo–1-phenylethyl 1–(3–fluorophenyl) cyclopentane–1–carboxylate) as a potent hit molecule (Figure 3B), which binds to the recombinant *Mtb* subunits δ with a dissociation constant (*K*_D_) of 20 µM [47]. DeMF1 inhibits NADH- and succinate-driven ATP synthesis of mycobacterial IMVs, as well as reconstituted F-ATP synthase, with an IC_50_ of about 0.5 µM [47]. As described for the mycobacterial subunits α, γ, and ε, the identification of a novel structural element in mycobacterial δ, which does not exist in its human counterpart, and its mechanistic role paved the way for a new compound target and the identification of the new inhibitor DeMF1.

### 2.5. The Fo-Domain Subunits a and c Represent Important Anti-TB Targets

**Figure 5:**
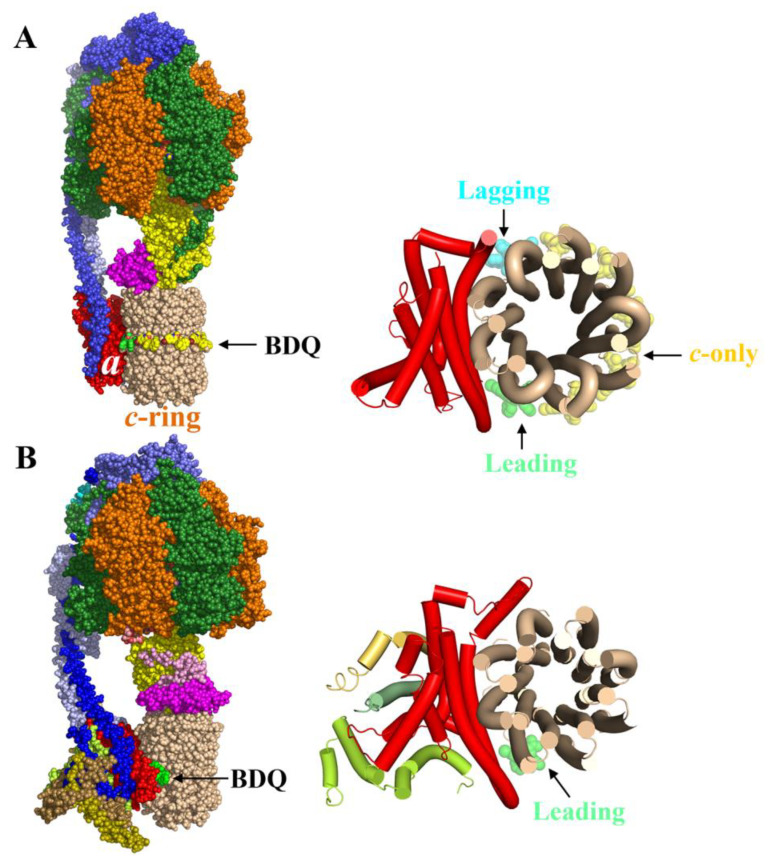
BDQ-bound *Mtb*- and human F-ATP synthase cryo-EM structure. (**A**) Side view of *Mtb* F-ATP synthase structure (PDB ID: 8j0S) [26]. Shown on right-hand side is the top view of *a*-*c_9_*-ring domain, highlighting one BDQ at the leading and lagging site of *a*-*c_9_*-ring interface, respectively. While five BDQ molecules were also seen at the *c*-only binding sites. (**B**) Human mitochondrial F-ATP synthase structure (PDB ID: 8ki3 [26]). The top view of the F_O_-domain, presented on the right-hand side, shows only one BDQ bound to the leading site.

The *Mtb c_9_*-ring translocates protons via E61 across the membrane using two half-channels in subunit *a*, and its revolution enables the F_1_-domain to carry out ATP formation. Therefore, interruption of the *c_9_*-ring rotation and/or the blockage of the proton-conducting half-channels are ideal inhibitor targets. This is nicely reflected in the binding mode of BDQ to the *Mtb* F_O_-domain, with five molecules bound to the *c*-only sites (Figure 5A), one at the so-called leading site, where a proton enters from the intermembrane space, and one BDQ molecule at the lagging site, where a proton will be released via the second half-channel into the cytoplasm [26]. Both the leading and lagging sites are formed by interfaces of subunits *a* and *c* (Figure 5A). BDQ’s dimethylamine (DMA) at the *c*-only sites is engaged in ionic intermolecular hydrogen bonding interactions with carboxyl side chain atoms of E61. Residues L59, A62, A63, Y64, F65, I66, and L68 from the adjacent *c*-subunits are involved in van der Waals interactions. Some of these interactions explain the observed BDQ-resistant clinical isolates with substitutions of the *c* subunit residues E61D, A63P, or I66M/V [48]. 

Importantly, BDQ binds to the detergent-isolated human mitochondrial F-ATP synthase with a *K*_D_ of 5.71 µM [26]. The BDQ-bound cryo-EM structure shows one BDQ sitting in the leading site, thereby inhibiting the rotation of the turbine (Figure 5B) [26]. BDQ’s 6-bromo-2-methoxyquinoline interacts with residues *c*F54, *c*A55, *c*A59, *c*L62, *a*I195, *a*L198, and *a*L202, the phenyl ring is anchored by residues *c*L62 and *c*L65, while the naphthyl ring makes π-π interactions with F63 [26]. BDQ inhibits ATP hydrolysis of the isolated human F-ATP synthase with an IC_50_ of 0.34 µM [26] and ATP synthesis of human microplasts with an IC_50_ of 0.34 µM [49], reflecting that BDQ’s binding to the leading site inhibits ATP synthesis and hydrolysis of the human enzyme.

#### 2.5.1. BDQ Derivatives 

**Figure 6:**
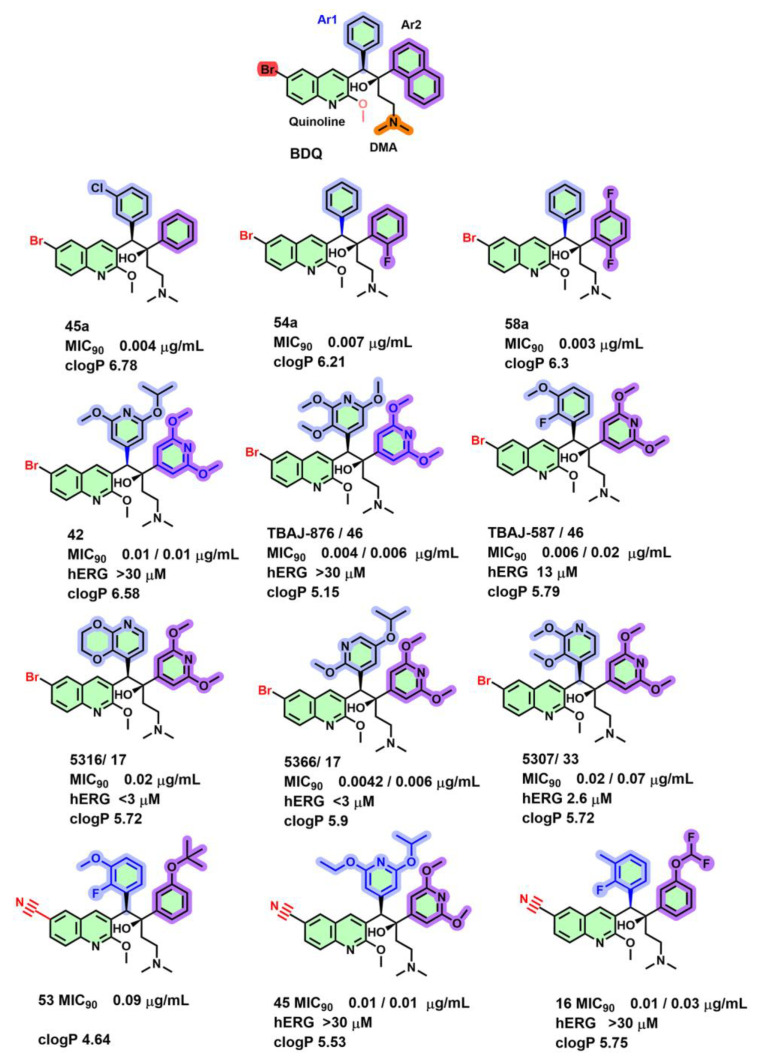
BDQ derivatives with their corresponding MABA/LORA TB MIC_90_.

Initial attempts to lower the lipophilicity of BDQ derivatives by varying the ‘Br’ at the sixth position on BDQ’s quinoline ring with –S-CH_3_, -SO_2_-CH_3_, -SO_2_-(CH_2_)_2_-NH_2_, -O-(CH_2_)_2_-NH_2_, -NH-(CH_2_)_2_-NH_2,_ CH=CH_2_, and cyanide (CN) groups have shown a reduction in MIC_90_ by more than 10-fold. Only the replacement of bromine with chlorine was tolerated, and it maintained an MIC_90_ activity comparable to BDQ [50,51]. These findings reveal that the Br- or Cl group on position six of quinoline and DMA (highlighted in red) are strictly required to maintain the MIC (Figure 6). Among the variations on the phenyl group (Ar_1_), only the 3-Cl group (**45a**) yielded an MIC_90_ of 0.004 µg/mL on *M. smegmatis* cultures (Figure 6) while replacing the naphthyl group with o-fluorophenyl (**54a**) or 2,5-difluorophenyl (**58a**) caused good potency (MIC_90_ of 0.007 and 0.003 µg/mL, respectively) [50,51], suggesting the tolerance for monocyclic aromatic rings.

Subsequent rounds of optimization on the Ar_2_ group with a panel of heterocyclic groups led to the identification of 3,5-dialkoxy-pyridine on the Ar_2_ position with lower lipophilicity and an MIC_90_ equivalent to BDQ [52,53]. However, this second series of molecules still retained human Ether-à-go-go-Related Gene (hERG) channel activity [52,53,54]. Further efforts were focused on varying the Ar_1_ group with multiple heterocyclic groups on BDQ to uncouple toxic hERG activity. Alkoxy substitution at the 3, 5 positions with a 4-aza atom on Ar_1_ (**42**, TBAJ-876, **cLogP 5.5** Figure 6) was required to lower hERG binding (>30 µM) and yet maintain their MIC_90_ in both replicating and non-replicating conditions [55]. Likewise, the analogs TBAJ-5316, -5366, and -5307 (Figure 6) with a benzo-dioxo, 2-methoxy-5-isopropoxy-3-aza-phenyl, or 2,3-dimethoxyo-pyrid-4-yl as an Ar_1_ group maintained MIC_90_ potency 0.02/0.02 µg/mL, 0.004/0.006 µg/mL, 0.02/0.07 µg/mL in the MIC_90_ assays, microplate alamar blue assay (MABA) and low oxygen recovery assay (LORA) [55]. These data highlight that reducing the lipophilicity, indicated by a lower cLogP, by substituting the Ar1/Ar2- with 3,5-dialkoxy-pyridyl groups afforded reduced non-specific hERG binding.

TBAJ-876 and its analogs showed a loss of MIC sensitivity to *c*D28, *c*E61, *c*A63, and *c*I66M mutants (MIC_90_ > 40 fold), confirming a similar mechanism of action as BDQ [56]. In addition, TBAJ-587 (**46**, Figure 6), presently in phase 2 studies, showed potency in both MABA and LORA assays with MIC_90_ 0.006 and 0.02 µg/mL, respectively. The analog revealed lesser hERG affinity with an IC_50_ of >10 µM and a tenfold lower *K*_D_ of 56.92 µM to the human mitochondrial F-ATP synthase compared with the parental molecule BDQ [26]. Further structure–activity studies on decreasing the lipophilicity found that replacement of Br with CN can also maintain its MIC potency depending on the presence of 2-F, 3-Me/3-OMe substituted phenyl (Ar_1_) ring and 3-OCHF_2_ group/3-O-C(CH_3_)_3_ substitutions on the Ar_2_ ring (analog **16**/**53**, Figure 6) [51,57]. Likewise, 2-ethoxy, 5-isopropoxy substituted pyridyl (Ar_1_) and 3,5-dimethoxy-pyridine (Ar_2_) (Figure 6 analog **45**) substitutions on 6-cyano-BDQ derivatives retained MIC_90_ potency [55]. Interestingly, among the cyano series of BDQ derivatives, only analog **16** [57] and **45** [55] (Figure 6) were equipotent on MABA/LORA (MIC_90_ with 0.01/0.03 and 0.01/0.01 µg/mL, respectively) and had reduced hERG binding (>30 µM).

#### 2.5.2. BDQ Combination Regimen Shortens TB Treatment Timeline

WHO guidelines on studies for finding a shorter MDR-TB treatment regimen by supplementing BDQ with first/second-line drugs have led to multicenter clinical trials [58,59,60,61]. The findings, comprising triple or quadruple drug combinations by including BDQ with the standard first line of treatment, have shown promise that the TB treatment timeline could be shortened to 60 days [60].

##### Triple Combination, Including Pretomanid, BDQ, Linezolid

Results from a recently concluded phase III clinical study (ZeNix, NCT02333799) highlighted that extensively drug-resistant (XDR) and pre-XDR participants (181) administered a three-drug regimen of BDQ (daily dose of 200 mg for 8 weeks, followed by 100 mg daily for 18 weeks), pretomanid (200 mg daily for 26 weeks), and linezolid (600 mg for 26 or 9 weeks) had favorable outcomes in 91% and 84% of cases, respectively [58]. The overall risk-to-benefit ratio indicated that the group receiving the three-drug combination with linezolid at 600 mg for 26 weeks showed a lower incidence of adverse events and fewer linezolid dose-related events in comparison to a 1200 mg dose cohort. Adverse events, such as peripheral neuropathy and myelosuppression, were associated with a 1200 mg dose of linezolid [58]. Likewise, in a multicenter randomized controlled clinical trial (referred to as New and Emerging Therapies for Drug-Resistant Tuberculosis (NExT)) assessing an all oral 6-month triple-drug regimen of levofloxacin, BDQ, and linezolid treatment for MDR-TB highlighted significantly favorable treatment outcomes in 51% of patients in comparison to standard first-line therapy [59].

##### Quadruple Combination, Including BDQ, Pretomanid, Moxifloxacin, and Pyrazinamide

The results of TB-PRACTECAL [60], a multicenter randomized phase 2b trial assessing the efficacy of BDQ, Pretomanid, Pyrazinamide (BPaZ) combination with/without moxifloxacin, recorded 89% favorable outcomes and showed superior bactericidal activity during the first 8 weeks (about 2 months) of treatment. Once daily dosing of BDQ (200 mg) had a similar bactericidal effect as that of the loading dose (400 mg daily for 14 days, followed by 200 mg daily for remaining days). The BDQ daily dose regimen combination showed a high percentage of negative sputum cultures, followed by the standard BDQ dosing regimen combination in comparison to control treatment of isoniazid (H), rifampicin (R), pyrazinamide (Z), and ethambutol (E) [60]. Treatment with BDQ, pretomanid, moxifloxacin, pyrazinamide (BPaMZ) in pyrazinamide-susceptible and rifamycin-resistant patients caused superior bactericidal effects by day 56 compared with the HRZE first-line treatment and highlighted the potential to shorten the treatment duration in drug-susceptible TB (DSTB). Further, this BPaMZ treatment has similar efficacy in HIV-positive and rifamycin-resistant patients. Elevation in peak liver enzymes was seen in DSTB patients treated with BDQ and pretomanid [60].

##### BDQ, Delamanid, Linezolid, and CFZ Combinations

A clinical study referred to as Building Evidence to Advance Treatment of TB (BEAT-TB) [61] from India with 158 MDR-TB patients and treatment with BDQ, delamanid, linezolid, and CFZ for 24–36 weeks found that 91% of patients had positive outcomes in fluoroquinolone-resistant (TBFQ+) cases, while the same regimen also resulted in a 69% positive MDR-TBSLI+ (second line injectables) resistant cases. Most of the adverse events were associated with CFZ-associated hyperpigmentation or linezolid-related anemia/myelosuppression and a low incidence of BDQ-related QT prolongation [61]. Phase 2 studies assessing the utility of CFZ in combination with BDQ, pretomanid, and pyrazinamide in the first 14 days of treatment showed no difference from standard therapy. CFZ had no measurable activity in the first 14 days and could not lower bacterial burden significantly in comparison to BDQ, pretomanid, and pyrazinamide combination or standard drug treatment [61].

## 3. Simplified BDQ Inspired Analogs

### 3.1. Quinoline Derivatives

**Figure 7:**
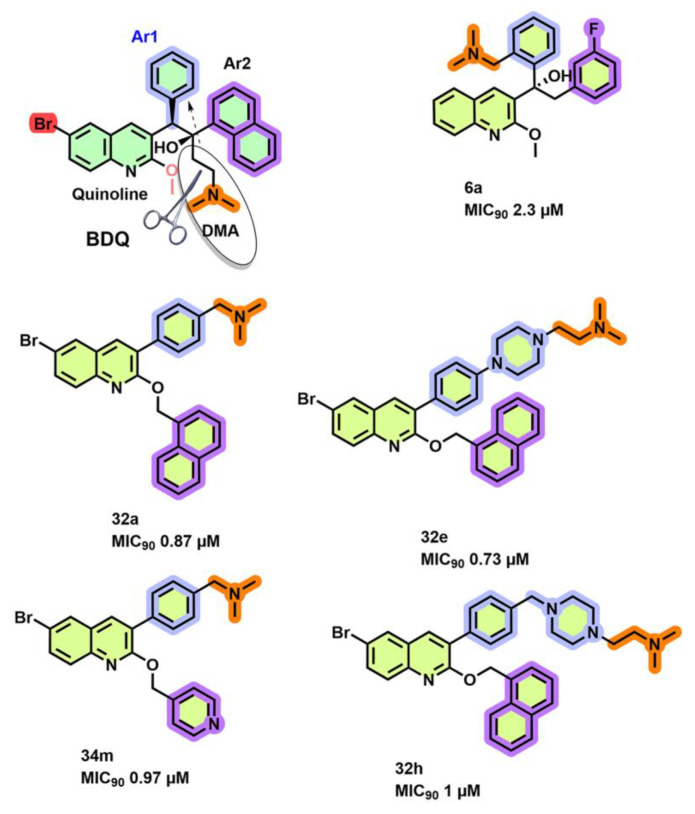
Simplified versions of BDQ analogs. The highlighted changes in DMAAr1/Ar2 substitutions within the BDQ scaffold maintain MIC_90_ but impair inhibition of ATP synthesis due to the loss of the diaryl pharmacophore group (blue and violet rings) and rearrangement of DMA (orange-colored atoms). Addition of the aryl Ar_2_ group to the 2-Meo group (**32a**, **32e**, **34m**, **32h**) did not improve MIC_90_ potency).

He et al. [62] attempted to simplify the chemical synthesis of BDQ by (i) removing chirality by re-arranging the DMA moiety (orange-colored atoms) on either of the Ar_1_/Ar_2_ ring substitutions (blue/violet-colored rings, respectively, Figure 7), (ii) removing the bromine/hydroxyl atoms, and (iii) modifying the Ar_2_ ring substitution onto the 2-OMe group and DMA moiety to Ar_1_ group. In one series, the DMA moiety is incorporated into the Ar_1_-ring, an m-fluoro-benzyl group is used as the Ar_2_-group, and the 6-bromine group is omitted to yield molecule 6a (Figure 7) with an MIC_90_ of 2.3 µM [62]. In another series, the best MIC_90_ active molecules were obtained from a series replacing the Ar_2_-ring with the 2-OMe group and the DMA onto the Ar_1_-group. Ligands **32a**, **32e**, **32m**, and **32h** (Figure 7) inhibited the *Mtb* H37Rv cultures with an MIC_90_ of 0.87, 0.73, 0.97, and 1 µM, respectively. Not surprisingly, with the removal of core pharmacophore features—chiral centers—aryl Ar_1_/Ar_2_ and the hydroxyl groups of BDQ, the compound **32a**, **32e**, **32m**, and **32h** showed reduced inhibition of ATP synthesis (IC_50_ of 20–40 µM). Furthermore, analogs **34m** and **34e** also maintained similar inhibition of growth with a two-fold increase in MIC_90_ potency towards drug-resistant clinical strains 12153, 6133 [62].

### 3.2. C5-Aryl Pyridine Modifications

**Figure 8:**
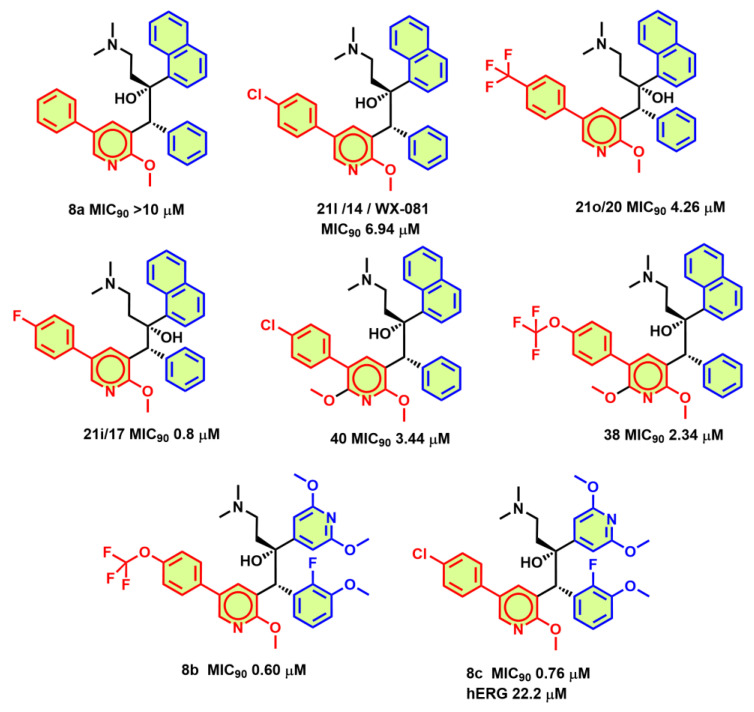
C5-aryl pyridine modification of the quinoline ring.

Studies from Barbaro et al. have shown that the replacement of the quinoline ring of BDQ with a 2-methoxy-pyridine group increased the MIC_90_ (>10 μM) [63]. Further medicinal chemistry efforts demonstrated that substitution of the phenyl group at position 5 of pyridine conferred reasonable potency (MIC_90_ < 10 μM). Further, SAR studies on the 5-phenyl-2-methoxy-pyridine to match the 6-bromo bio-isostere group on quinoline improved cell growth inhibition. Substitutions such as 4-Cl, 4-CF_3,_ and 4-F on the 5-phenyl-2-methoxy-pyridine (Figure 8) drastically improved the MIC_90_ on *Mtb* H37Rv cultures to 6.94, 4.26, and 0.8 μM, respectively [63].

Ding et al. [64] worked on phenyl-2-methoxy-pyridine scaffold derivatives and selected WX-081 as their lead candidate, which is in phase 2 studies. Excellent anti-mycobacterial properties in vitro and in vivo of WX-081 could be observed [64]. The medical chemistry efforts demonstrated that 4-Cl (WX-081/Sudapyridine, Figure 8), as well as 4-OCF_3_ substitutions on the 5-phenyl-2-methoxy-pyridine derivative of BDQ, were almost equipotent as BDQ in both MABA/LORA assays with MIC_90_ of 0.08/0.56 μg/mL and 0.03/0.61 μg/mL, respectively. WX-081 inhibited the growth of MDR-TB (MIC_90_ = 0.47 μg/mL) and DS-TB (MIC_90_ = 0.15 μg/mL) strains. Furthermore, absorption–distribution–metabolism–excretion–toxicity (ADMET) studies and the evaluation of the pharmacokinetic parameters in CD-1 mice suggested low cell permeability and high plasma binding, moderate clearance in mouse liver microsomes, and lesser hERG activity (IC_50_ > 30 μM). WX-081 was also assessed for anti-TB efficacy in acute and chronic TB infection models. In an acute infection model, WX-081 and BDQ were administered at three doses (5, 10, 23 mg/kg) and rifampicin at 15 mg/kg for 5 consecutive days per week for a total of 4 weeks. WX-081 treatment results in a remarkable reduction in bacterial load in mouse lungs. At 5 mg/kg, bacterial load was reduced by over 3 log units at day 38, resembling BDQ/rifampicin. In a chronic TB infection model, both WX-081 and BDQ at 5 mg/kg and 20 mg/kg afforded a reduction in bacterial load by 1 log CFU and 2.5 log CFU reduction [64].

Barbaro et al. have further explored similar substitutions on the 5-phenyl-2-methoxy-pyridine ring in the BDQ and TBAJ-587 scaffold [65]. 4-Cl (**8c**), 4-F (**17**), and 4-CN (**8l**) substitutions on the 5-phenyl-2-methoxy-pryidine ring of TBAJ-587 afforded more potent MIC_90_ of 1.9, 4.3, and 6.0 μM, respectively, in comparison to the quinoline ring of BDQ (**8a**, **14**, **17**, **20**, **38**, and **40** Figure 8). Characterization of the diastereomeric pairs demonstrated that RR and SS had only µM MIC_90_ potency, while RR, SS diastereomers of 4-OCF_3_ (**8b**), 4-Cl (**8c**) (Figure 8), 4-CN (**8l**) substituted 5-phenyl-2-methoxy-pryidene had an MIC_90_ of 1.09, 1.45, and 2.25 μM, respectively. Furthermore, separation of the enantiomers highlighted that only S,S enantiomers of 4-OCF_3_, 4-Cl, 4-CN derivatives had sub-µM potency on *Mtb* H37Rv cultures with an MIC_90_ of 0.60, 0.76, 0.98 μM, respectively. Among the 4-OCF_3_ (8b) and 4-Cl (**8c**) derivatives (Figure 8), the 4-chlorophenyl pyridyl derivative (**8c**) not only showed lesser affinity towards the hERG channel with an IC_50_ of 22 μM but also retained MIC_90_ potency. [65].

### 3.3. Squaramide Derivatization

**Figure 9:**
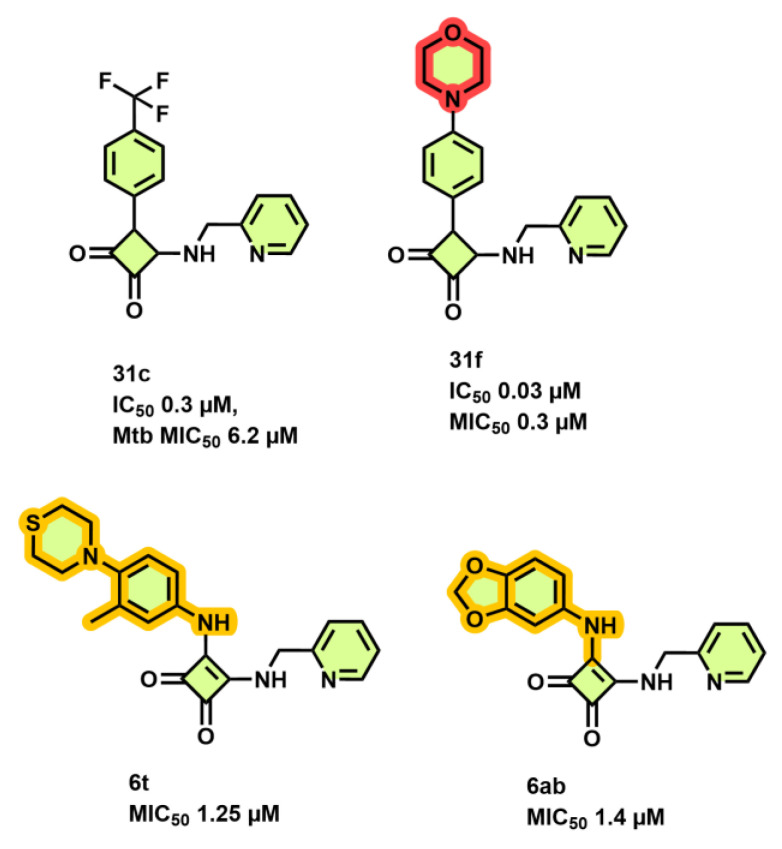
Squaramide derivatives.

Tantry et al. [66] reported a novel scaffold squaramide (**31c**, Figure 9) that binds to a subunit *a-c* interface, which is different from that bound by BDQ and inhibits ATP synthesis with an IC_50_ of 0.3 µM and *Mtb* growth with a MIC_50_ of 6.2 µM [66]. Selection for spontaneous resistance (**31f**, (Figure 9)) yielded the two mutants, K179N on subunit *a* and D28N on subunit *c*, confirming the involvement of an *a-c* interface. SAR results revealed that the morpholine ring (Figure 9, shown in magenta) at position 4 of the phenyl moiety yielded the best IC_50_ (0.03 µM) and MIC_50_ (0.3 µM) values when compared with the parent compound (**31c**, Figure 9). However, smaller substitutions, such as a CN group on the phenyl moiety, eroded the inhibitory effects compared with CF_3_ substitution. Substitutions on the “amino-methyl-2-pyridine” moiety revealed that the 2-aza atom on the pyridyl moiety was essential for activity. Varying the pyridine moiety with pyrazine or phenyl showed a decreased and total loss of activity, respectively. Extending the substitution at methyl atoms of amino-methyl-2-pyridine or the aryl character at position 5 of the pyridine did not improve the activity profile in comparison to the parent compound [66]. Another study demonstrated that diamino-substituted cyclobut-3-ene-1,2-dione derivatives **6t, 6ab** (MIC_50_ 1.25 and 1.4 µM, respectively, Figure 9) were also able to maintain their anti-TB potency on H37Rv *Mtb* cultures as well as multi-drug resistant (MDR)–13946, 14862 TB strains [67].

### 3.4. Amiloride Derivatives

**Figure 10:**
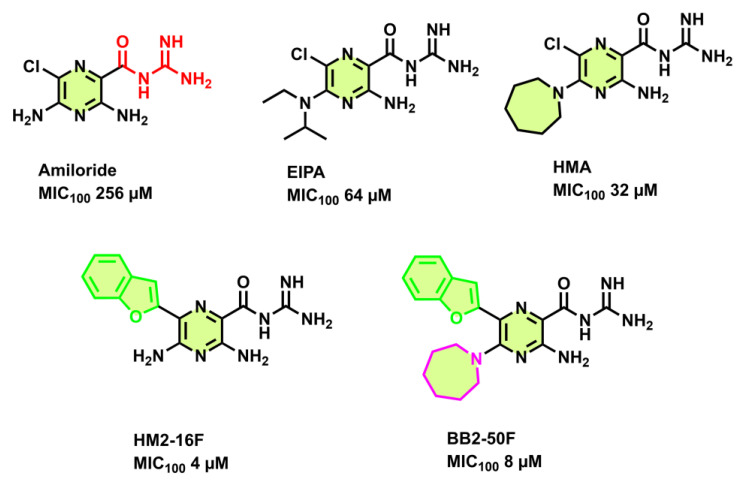
Amiloride derivatives.

Based on bioisosterism to BDQ, amiloride, 5-(N, N-hexamethylene)-amiloride (HMA), and EIPA (5-(N-ethyl, N-isopropyl)-hexamethylene amiloride) (Figure 10) were able to inhibit the growth of *Mtb* H37Rv with a weak potency of 256 µM, 32 µM, 64 µM, respectively [68]. Amiloride derivatives also inhibited ATP synthesis in *M. smegmatis* IMVs. HM2-16F, a derivative of the FDA-approved drug amiloride, inhibits *Mtb* H37Rv cultures as well as BDQ-resistant mutants. HM2-16F (Figure 10) binds weakly to the *Mycobacterium phlei* F-ATP synthase *c*-subunit [68] and displayed moderate bactericidal activity (MIC_100_ 4 µM) towards *Mtb* cultures. Interestingly, HM2-16F lacked cross-resistance to F-ATP synthase *c* subunit A63P mutation or to Rv0678 (MMP5-MMPl5 efflux pump). The compound was described as a dual inhibitor, working in concert with a mycobacterial cytochrome *bd* oxidase inhibitor [68]. 

Further medicinal chemistry on this scaffold by this group resulted in the lead molecule BB2-50F (MIC_100_ of 8 μM) with an azepanyl group added onto the fifth amino group (Figure 10, magenta highlighted group) [69]. The compound retained its potency against a variety of *Mtb*-resistant strains. Interestingly, BB2-50F at 10× its MIC concentration was more potent (>6 log_10_ reduction in CFU/mL) of hypoxia *Mtb* mc^2^ 6230 cultures [69]. Engineering a 5-(N, N-hexamethylene) substituent onto BB2-50F, which illuminates the target protein upon covalent binding/cross-linking, visualized the dual targeting of the compound to the mycobacterial Sdh-1 and F-ATP synthase [69]. Transcriptional profiling studies also highlighted that BB2-50F (0.03–30× MIC) influences the reactive oxygen species (ROS) pathway in mediating its killing actions in aerobic growth conditions [69].

### 3.5. High Throughput Hits

**Figure 11:**
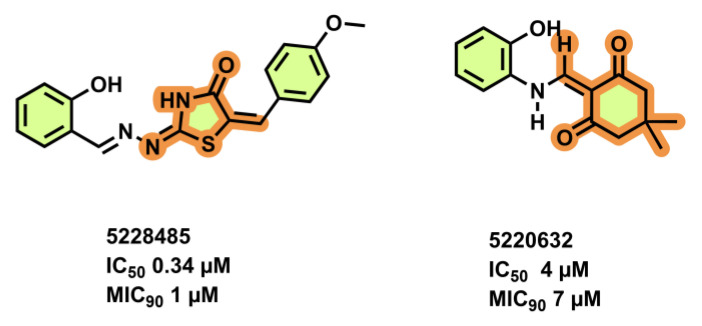
HTS hits, inhibiting the mycobacterial F-ATP synthase.

High throughput screening (HTS) of 700 anti-TB compounds (from CSIR-IIIM repository library) on *M. smegmatis* IMVs identified the two potent compounds 5228485 and 5220632 (Figure 11), which inhibited ATP synthesis (IC_50_ = 0.34 µM and 4 µM, respectively), as well as *Mtb* H37Rv growth (MIC_90_ = 1 µM and 7 µM, respectively), and showed µM-efficacy in J774 macrophages (MIC_50_ = 4 and 8 µM, respectively). Spontaneous-resistant mutants at subunits *a* and *c* indicated the F_O_-domain as the target [70].

### 3.6. Disubstituted Tetrahydroisoquinoline Derivatives 

**Figure 12:**
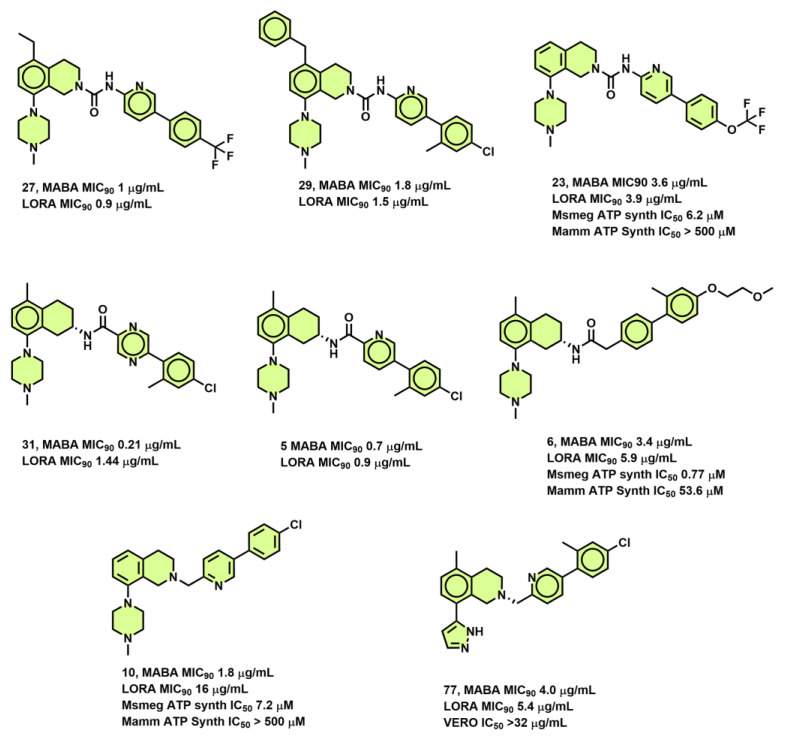
Tetrahydroisoquinoline (THI) derivatives.

Studies from Lu, G. L. et al. [71] show that 5,8-disubstituted tetrahydroisoquinoline (THI) derivatives are effective inhibitors of the *Mtb* H37Rv strain. Compounds such as **27**, **29** (Figure 12) from this series revealed equipotency in both the MABA and LORA assays with MIC_90_ of 1, 0.9 µg/mL and 1.8, 1.5 µg/mL, respectively. Compounds **23** and **10** (Figure 12) displayed higher selectivity > 500 µg/mL for *M. smegmatis* to human ATP synthesis. The authors suggest that bulky groups such as benzyl are tolerated at position 5 of THI, while N-methyl-piperazine was most preferred at position 8 of THI [71]. The same group also explored the tetrahydronaphthalene amide derivatives as potent inhibitors of the *Mtb* H37Rv strain. Compounds **31** and **5** (Figure 12) are almost equipotent in both MABA and LORA assays with an MIC_90_ of 0.21 and 1.44 µg/mL, respectively. Compound **77** showed lower hERG inhibition with an MIC_90_ of 4.0 µg/mL in the MABA assay. In comparison, compound **6** was shown to have a 70-fold selectivity in *M. smegmatis* vs. human ATP synthesis [72].

### 3.7. Amodiaquine Derivatives

**Figure 13:**
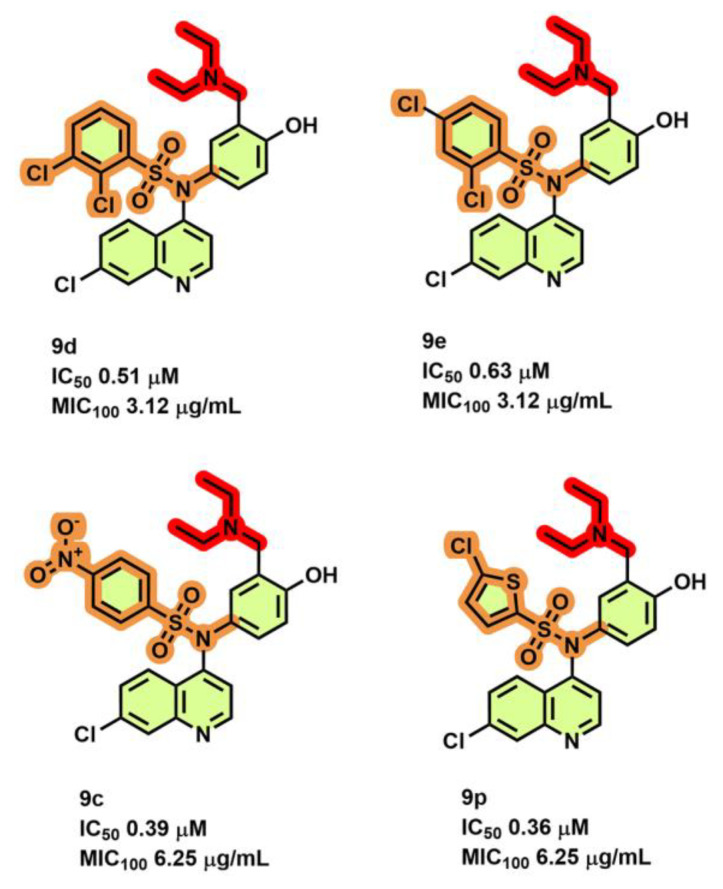
Amodiaquine derivatives.

Singh et al. [73] performed a structure-guided lead optimization approach on their hit molecule—amodiaquine—and derivatized four ligands **9c**, **9d**, **9e**, and **9p** (Figure 13). The analogs of these four ligands showed sub-µM potency with an IC_50_ of 0.39, 0.51, 0.63, and 0.36 µM in ATP synthesis inhibition in *M. smegmatis* IMVs. Among this series, analogs **9d** and **9e** were the most potent in *Mtb* H37Rv growth inhibition (MIC_100_ = 3.12 µg/mL) when compared to analogs **9c** and **9p** (MIC_100_ at 6.25 µg/mL). Interestingly, **9d** showed a higher selectivity index of >96-fold towards *Mtb* over Vero cells, bactericidal activity on hypoxic cultures at 100 µg/mL (2.3 log10 CFU/mL), and a low clearance rate of 0.06 L/h/kg. In vivo mycobacterial efficacy determined using a chronic mouse infection model suggested that **9d** at a dose of 173 µmol/kg conferred a survival of 60%, while the smaller dose could protect only 40% of the mice tested. In comparison, there was a 90% survival of mice with INH (182 µmol/kg). A reduction in bacterial load of 2.12 log_10_ CFU/mL and 1 log_10_ CFU/mL was seen in lung and spleen tissues for high and low doses used, respectively [73].

## 4. Cross Resistance: Necessity of Combination Therapies for Synergy

**Figure 14:**
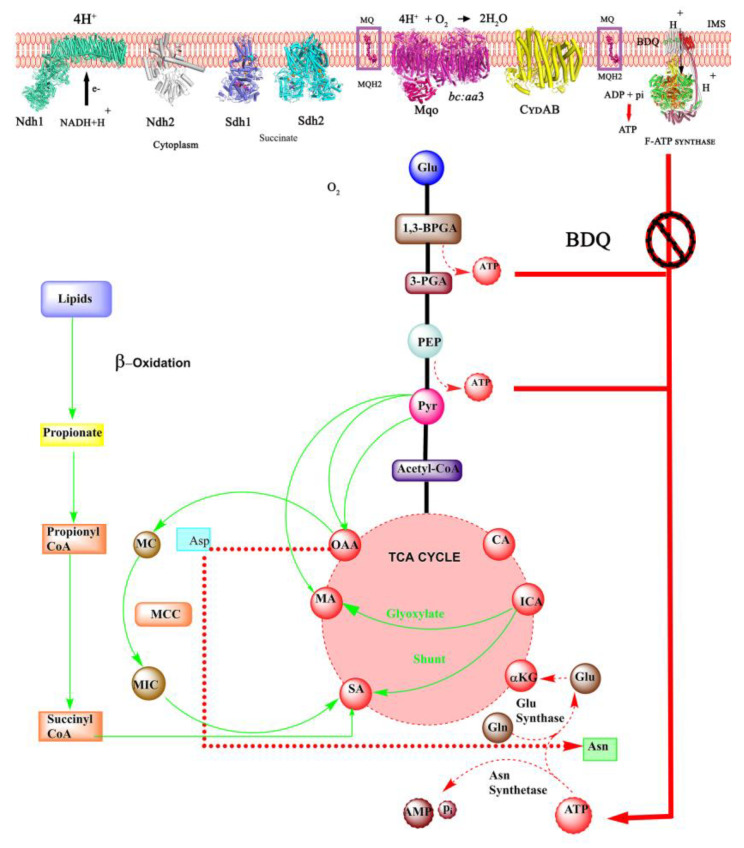
BDQ actions on the energy metabolism of *Mtb*. The pathogen activates β-oxidation, the methyl citrate cycle, and the glyoxylate shunt pathways highlighted in green to compensate for ATP depletion. However, with depleting levels of ATP, the energy-dependent Gln synthase activity co-lapses after a few cycles of growth, leading to death.

Interestingly, the works of Mackenzie et al. highlighted the possibility of combination therapies that can target both OXPHOS and glycolysis [74] and confer rapid killing of *Mtb*. To compensate for the loss of ATP by BDQ treatment, *Mtb* induces pleiotropic effects on its central metabolism by enhancing respiration through the glyoxylate shunt pathway/β-oxidation of fatty acids via the methyl citrate cycle (MCC), thereby generating substrates for the TCA cycle [74]. With declining ATP levels due to BDQ treatment, a few cycles of this pathway would consume the depleting basal ATP and eventually implode into a collateral loss of glutamine synthetases [75] needed for regenerating substrates of the TCA cycle (Figure 14), thereby pushing *Mtb* to a metabolically lethal state [74].

Network analysis showed that upon administration of BDQ, mycobacteria can rewire their energy metabolism by activating transcription factors such as Rv0324 (thiosulfate sulfur transferase) and Rv0880 (uncharacterized transcription factor) to abrogate the cidal effects of the inhibitor [76]. Knockout of transcription factors Rv0324 and Rv0880 significantly enhanced the bactericidal effects of BDQ (15 μM) by 6- and 22-fold lower growth compared with H37Rv cultures, respectively [76]. A network analysis on anti-TB drugs that could lower Rv0880 levels has successfully led to the identification of pretomanid (Table 1) as a combination drug, with BDQ showing additive-to-mild synergistic effects. Conversely, Rv0880 overexpression has eliminated the synergy of this drug combination [76]. Pretomanid is now FDA approved in combination with BDQ and linezolid for the treatment of XDR-TB [77].

**Figure 15:**
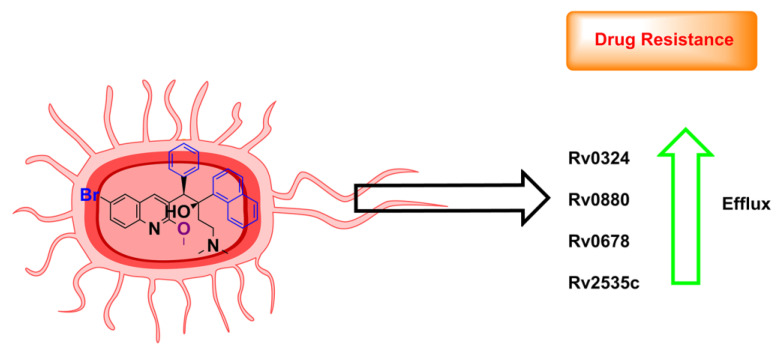
*Mtb* response on exposure to BDQ. *Mtb* activates expression of Rv0324 and Rv0880. Rv0678 and Rv255C lead to elevated drug efflux activity and drug resistance.

Non-target-based mutations in Rv0678 putatively increase BDQ/CFZ efflux through the MmpS5-MmpL5 pump [78,79]. Mutations in the transcriptional regulator Rv0678 (mmpS5) elevated the expression levels of MMPL5 and enhanced efflux activity (Figure 15). Similarly, loss-of-function mutations in pepQ (Rv2535c), encoding a Xaa-Pro aminopeptidase [80], displayed a loss of MIC sensitivity to BDQ in comparison with the WT H37Rv strain [80]. Though the underlying mechanism for pepQ mutation is yet to be clarified, overexpression of pepQ renders susceptibility to BDQ. Co-incubation with efflux inhibitors such as verapamil or reserpine (Table 1) [81] demonstrated additive actions, leading to enhanced killing of mycobacteria in both WT and mutant cultures. However, verapamil did not elicit similar synergy regarding bactericidal activity, nor did it avert the efflux pumps when tested in vivo in mice [81]. Nevertheless, from a mechanistic point of view, these studies highlight the utility of MmpL5 or pepQ inhibitors in combination with BDQ as an attractive strategy to delay the emergence of BDQ resistance [80,82].

**Figure 16:**
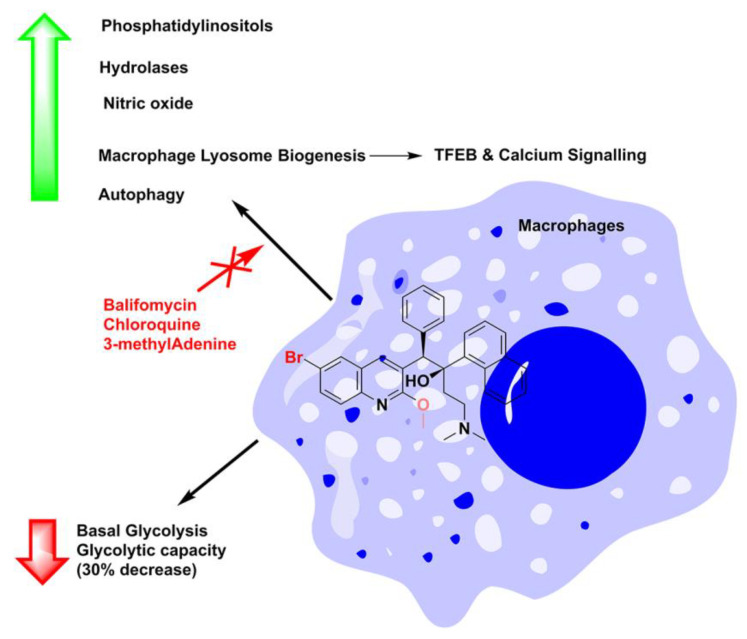
BDQ-mediated actions on host macrophages. Elevation of nitric oxide levels, phagosome–lysosome fusion, and autophagy to downregulate the glycolysis and glycolytic capacity in host macrophages.

BDQ treatment induced significant metabolic reprogramming of both *Mtb*-infected and resting macrophages. The drug-altered host metabolism by downregulating basal glycolysis and glycolytic capacity by 30% and by elevating glucose/phosphatidylinositol lipid levels in comparison with untreated cells. BDQ was able to activate a plethora of defense mechanisms that led to the overexpression of nitric oxide levels, phagosome–lysosome fusion/autophagy-related genes, such as transcription factor-E Box (TFEB), and calcium signaling (Figure 16), leading to rapid intracellular killing of mycobacteria. Several lines of study have shown that *Mtb* selectively modulates autophagy flux in macrophages, prevents phagosome maturation, and evades lysosome targeting, thereby enabling survival [83,84]. BDQ-treated macrophages showed better killing of mycobacteria in comparison to other anti-TB drugs. Taken together, these findings highlight the possibility of including autophagy inducers (known molecules such as rapamycin, EGCG, and others [44,85]) in a dual-therapy combination regimen with anti-TB drugs to promote autophagy and enable faster *Mtb*-killing [83,86].

Likewise, *Mtb* has the ability to rapidly re-route its ETC, enhancing respiration by switching to alternative terminal oxidases, such as cytochrome *bd* oxidase, to maintain ATP homeostasis. Overexpression of CydAB seems to positively regulate the gene levels of atpA, atpG, atpC, and atpF, while the knockout of CydAB genes reduces the levels of all four genes by 2.4 to 2.8-fold in comparison with WT strains [87]. This phenotype could explain why cytochrome oxidase *bd* knockout strains are hypersensitive to BDQ [88]. Not surprisingly, treatment of BDQ (8 μg/mL) in a cytochrome *bd* knockout strain enhances bacterial killing by 55%. Additionally, ΔCydAB strains were hypersensitive to Q203 [28] and CFZ (Table 1) [89]. Furthermore, enhanced respiration as a consequence of Q203/BDQ treatments leads to increased ETC activity, which, in turn, potentiates the production of ROS by CFZ. Overexpression of the CydA gene can partially restore the growth [88]. This evidence clearly points to the utility of the combination therapy of CFZ, BDQ, and ND-011992 [90], a cytochrome *bd* oxidase inhibitor, in the enhanced killing of *Mtb* [89,90].

Assessing the metabolic lethality of depleted glutamine levels, Wang et al. used a non-specific inhibitor of Gln synthetase, methionine sulfoximine (Table 1), to show a dose-dependent growth inhibition of *Mtb* [75]. This growth inhibition was rescued with an exogenous addition of glutamine, further corroborating the critical influence of glutamine levels on mycobacterial growth. Synergistic effects of methionine sulfoximine (0.25 x MIC_50_) with BDQ showed a further 20-fold decrease in BDQ MIC. This synergistic effect could be abrogated by elevating glutamine levels externally. Contrastingly, glutamine addition did not change the MIC effects of BDQ, suggesting glutamine synthetase as a critical downstream secondary target-enhancing BDQ potency. Furthermore, the synergistic effects were also seen with the selective GlnA1 synthetase inhibitor having a trisubstituted imidazole derivative (2,4,5-TIM, Table 1), suggesting the potential utility of GlnA1 inhibitors in combination with BDQ for potent *Mtb* killing [66].

## 5. Conclusions/Future Perspectives

The presence of non-canonical mechanisms of regulation in ATP synthase activity is mediated by selective epitopes in mycobacterial F-ATP synthases. The described selective epitopes are not seen in the host enzyme and have been exploited by structure-based drug discovery with successful hit identification of GaMF1 [37], EGCG [42], AlMF1 [33], EpNMF1 [40], DeMF1 [47], and the lead GaMF1.39 [38]. Interestingly, recent evidence points to the presence of upstream target(s) that positively regulate *Mtb* F-ATP synthase levels. Knockdown of upstream targets such as *ptpA* lowered *Mtb* F-ATP synthase subunit α protein levels [34]. This finding presents us with an arsenal to explore novel mechanistic inhibitors that can negatively regulate *Mtb* F-ATP synthase activity. As there is a dearth of ligands that selectively target the catalytic β-subunits or non-catalytic α-subunits in mycobacteria, such upstream target inhibition could pave the way for inhibitor development without target toxicity concerns in the host. Strikingly, the illustration that sgRNA-mediated transcriptional inhibition afforded much greater killing of mycobacteria than the clinical anti-TB drug BDQ might provide an avenue of precision medicine by targeting the mycobacterial atpE genes and could open an avenue for an mRNA vaccine or an antibody-based approach [29]. Understanding the reconfiguration of *Mtb* respiratory metabolism to offset the effect(s) of anti-mycobacterial drugs would help us to target alternative respiratory or metabolic pathways common to both anaerobic and aerobic growth, such as glycolysis. As *Mtb* employs β-oxidation, the methyl citrate cycle, glyoxylate, and shunt pathways to replenish declining energy levels, the approach of glycolytic inhibitors or glutamine synthase inhibitors could enhance the lethality of anti-mycobacterials and thwart the emergence of drug resistance [75]. With increasing atomistic-level insights into the targets of anti-TB inhibitors, their genetic, metabolic, and combinatory effects, the increasing variety of BDQ-inspired and squaramide analogs, as well as the results emerging from clinical phase studies of BDQ in combination with first-line drugs, there is reason to be optimistic that potent drug combinations will provide a cure for MDR- and XDR-TB.

## Figures and Tables

**Table 1:** Standard TB drugs/ligands tested in combination with BDQ.

Name	Structure
Isoniazid	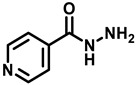
Rifampicin	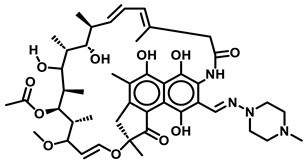
Pyrazinamide	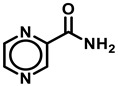
Reserpine	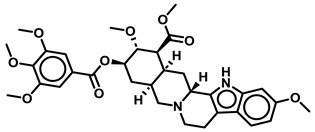
Verapamil	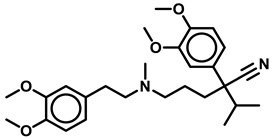
Methionine Sulfoxime (MSO)	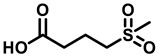
2,4,5-TIM	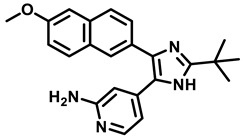
Pretomanid	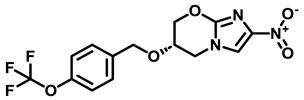
Clofazimine	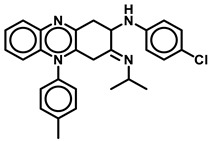
Q203	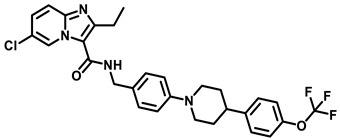
ND-011992	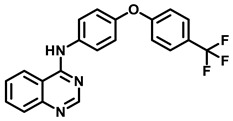
Delamanid	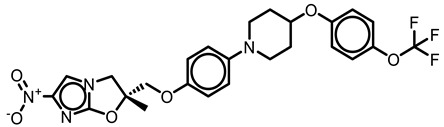

## Abbreviations

ADMET	Absorption–Distribution–Metabolism–Excretion–Toxicity
ADP	Adenosine Diphosphate
AlMF1	Alpha-Mycobacterial F_1_-ATPase
ATP	Adenosine Triphosphate
BDQ	Bedaquiline
BEAT-TB	Building Evidence to Advance Treatment of TB
BPaZ	BDQ, Pretomanid, Pyrazinamide Combination
CFU	Colony-Forming Unit
CFZ	Clofazimine
CRISPR	Clustered Regularly Interspaced Short Palindromic Repeat
C-terminal Domain	CTD
DeMF1	Delta mycobacterial F_1_-ATPase
DMA	Dimethylamine
DSTB	Drug-Susceptible TB
EGGC	Epigallocatechin Gallate
EIPA	5-(N-ethyl, N-isopropyl)-hexamethylene Amiloride
EpMF1	Epsilon Mycobacterial F_1_-ATPase
EpNMF1	Epsilon-N-terminus Mycobacterial F_1_-ATPase
ETC	Electron Transport Chain
GaMf1	Gamma Mycobacterial F_1_-ATPase
hERG	Human Ether-a-go-go-Related Gene
HMA	5-(N, N-hexamethylene)-amiloride
IMVs	Inverted Membrane Vesicles
INH	Isoniazid
LORA	Low Oxygen Recovery Assay
MABA	Microplate Alamar Blue Assay
MCC	Methyl Citrate Cycle
MDR-TB	Multidrug-Resistant TB
MIC	Minimal Inhibitory Concentration
MQ	Menaquinone
MQH2	Menaquinol
*Mtb*	*Mycobacterium tuberculosis*
NADH	Nicotinamide Adenine Dinucleotide Hydrogen
NDH	NADH Dehydrogenase
NExT	New and Emerging Therapies for Drug-Resistant Tuberculosis
OXPHOS	Oxidative Phosphorylation
Pi	Phosphate
pmf	Proton motive force
PUP	Prokaryotic Ubiquitin-like Protein
PZA	Pyrazinamide
ROS	Reactive Oxygen Species
Sdh	Succinate Dehydrogenase
SLI	Second Line Injectable
Sq	Squaramide
TB	Tuberculosis
TBAJ	TB Alliance-Janssen
TBFQ+	Fluoroquinolone-Resistant TB
TCA	Tricarboxylic Acid
TFEB	Transcription Factor-E Box
THI	Tetrahydroisoquinoline
XDR	Extremely Drug Resistant
